# O‐Arm Navigation Enhances Facet Preservation Without Compromising Clinical Outcomes in UBE Decompression for Radiographically Stable Adult Degenerative Scoliosis: A Single‐Center Comparative Study

**DOI:** 10.1111/os.70315

**Published:** 2026-05-08

**Authors:** Yi Liu, Yiwei Xie, Zhibao Chen, Ruijun Xu, Haojie Chen, Xiaojian Ye, Jiangming Yu

**Affiliations:** ^1^ Department of Orthopedics, Tongren Hospital Shanghai Jiao Tong University School of Medicine Shanghai China; ^2^ Center for Spinal Minimally Invasive Research Shanghai Jiao Tong University Shanghai China; ^3^ Shanghai Key Laboratory of Flexible Medical Robotics, Tongren Hospital, Institute of Medical Robotics Shanghai Jiao Tong University Shanghai China

**Keywords:** biportal, endoscopy, facet joint, scoliosis, spinal stenosis, surgical navigation

## Abstract

**Objective:**

In radiographically stable adult degenerative scoliosis (ADS), unilateral biportal endoscopic (UBE) decompression alone is effective in alleviating symptoms; however, executing adequate decompression without excessive facetectomy in rotated, tortuous anatomy remains challenging. Intraoperative O‐arm navigation has the potential to enhance procedural accuracy of UBE decompression. This study compared the clinical outcomes and radiological parameters between O‐arm navigation–assisted and conventional fluoroscopy‐guided UBE decompression alone in stable ADS.

**Methods:**

This single‐center retrospective study included 63 patients with radiographically stable ADS who underwent UBE decompression alone between 2021 and 2023 (navigation, NAV: *n* = 34; non‐navigation, NON‐NAV: *n* = 29). This study presents details about patients' demographics, perioperative parameters, and up to 24 months follow‐up outcomes. Primary endpoint was the facet preservation rate (FPR) at 1‐month post‐operation, quantified by CT‐based 3D volumetry. Secondary endpoints included DCSA, lateral recess height/angle, dynamic angulation/slip, patient‐reported outcomes (VAS/ODI), and complications. Data were analyzed using independent t‐tests, Wilcoxon rank‐sum tests, and repeated‐measures ANOVA as appropriate.

**Results:**

Operative time and length of stay were slightly shorter in NAV but not statistically different; estimated blood loss was comparable. NAV and NON‐NAV groups showed significant improvements in VAS of leg/back pain and ODI at 1 month and last follow‐up, without between‐group differences. DCSA increased substantially in both groups (~200%–250%); but dispersion was smaller in NAV, indicating more uniform decompression. Structural preservation favored NAV (higher residual lamina‐facet volumes). Segmentally, NON‐NAV exhibited greater increases in dynamic angulation (8.5° ± 1.2° vs. 6.2° ± 1.4°, *p* < 0.001) and early slip (2.8 ± 0.8 vs. 1.8 ± 0.8 mm, *p* < 0.001), although radiographic instability thresholds were not exceeded. Global sagittal and coronal parameters were largely comparable between groups over time.

**Conclusion:**

In stable ADS, O‐arm navigation for UBE decompression did not prolong operative time nor increase blood loss, and yielded tighter boundary control of decompression, higher facet preservation, and smaller segmental perturbations, while maintaining equivalent symptomatic improvement. The value of O‐arm navigation lies in enabling precise and sufficient decompression while limiting medial facetectomy within stability‐preserving margins.

## Graphical abstract

O‐arm navigation–assisted unilateral biportal endoscopic decompression achieves precise and sufficient neural decompression in stable adult degenerative scoliosis, enhancing facet preservation and reducing segmental motion changes while maintaining comparable symptomatic improvement to conventional fluoroscopy, demonstrating the value of navigation in stability‐preserving minimally invasive spine surgery.

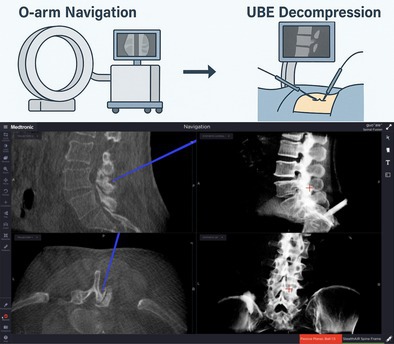

**Abbreviations**
ADS: adult degenerative scoliosis
CT: computed tomography
DCSA: dural sac cross‐sectional area
DT: dural tear
FPR: facet preservation rate
IRB: Institutional Review Board
LL: Lumbar lordosis
MRI: magnetic resonance imaging
NAV: navigation group
NON‐NAV: non‐navigation group
ODI: oswestry disability index
PI: pelvic incidence
PSIS: posterior superior iliac spine
UBE: unilateral biportal endoscopy
VAS: visual analogue scale

## 1 Introduction

Adult degenerative scoliosis (ADS) is an acquired deformity characterized by multilevel degeneration, axial rotation, and coronal/sagittal imbalance. It is frequently associated with multi‐compartmental stenosis of the central canal, lateral recess, and neural foramina, which clinically presents as neurogenic claudication, radicular pain, and axial low back pain [1, 2, 3]. Systematic reviews indicate that the burden of ADS and adult spinal deformity increases with age, posing sustained challenges to quality of life and healthcare resources [1, 2]. In ADS with dynamic stabilization, decompression alone as a symptom‐directed strategy has been considered to be a reasonable surgical option [4].

Benefiting from minimal soft‐tissue disruption, unilateral biportal endoscopy (UBE) allows ipsilateral and over‐the‐top contralateral decompression through a single approach and has gradually become a mainstream minimally invasive treatment for degenerative lumbar stenosis [5, 6, 7]. However, within the rotated and torsional anatomy of ADS, conventional UBE guided by anatomic heuristics and stepwise fluoroscopy still encounters three methodological challenges: (i) uncertainty in intraoperative localization and spatial orientation; (ii) limited controllability of decompression boundaries (ipsi/contralateral lamina, lateral recess, foramen, and facet joint), predisposing to inadequate or excessive facetectomy; and (iii) a steep learning curve with heavy reliance on fluoroscopy, which is closely associated with dural tears and residual stenosis [5, 6, 7, 8, 9].

Intraoperative O‐arm based navigation offers a verifiable solution to these difficulties. Through a closed‐loop workflow of intraoperative O‐arm scanning, registration, and real‐time spatial feedback, navigation marks out critical osseous and corridor anatomy and reduces dependence on repeated fluoroscopy and subjective landmarks, thereby improving precision and consistency in complex anatomy. Concurrently, multiple studies and reviews have suggested potential reductions in staff radiation exposure and improvements in surgical efficiency by using navigation [10, 11, 12]. In UBE settings, recent studies indicate that integrating O‐arm navigation with UBE can enhance working‐corridor accuracy, and in selected contexts decrease operative time or team radiation exposure [13, 14, 15, 16]. However, evidence remains limited on whether navigation can improve decompression precision while preserving the facet joint within stability‐preserving margins during UBE decompression alone for stable ADS—an evidence gap that motivated the present comparative study.

Therefore, we conducted a single‐center retrospective cohort study in radiographically stable ADS to evaluate O‐arm navigation–assisted UBE decompression alone with respect to decompression precision, segmental alignment, and stability, compared with conventional fluoroscopy‐guided UBE decompression alone. We hypothesized that navigation would improve boundary control and facet preservation without compromising clinical improvement.

## 2 Materials and Methods

### 2.1 Study Design and Patients

Sixty‐three Patients were consecutively enrolled if they met the inclusion and exclusion criteria and completed follow‐up during the study period (2021–2023). Radiographic intervertebral stability (slip < 3 mm, angular change < 10°) was a prerequisite for all included cases with no clinical indication for fusion, in line with previously reported radiographic thresholds for lumbar stability.^17^ According to the usage of intraoperative navigation, patients were assigned to a navigation group (NAV, *n* = 34) or a non‐navigation group (NON‐NAV, *n* = 29). The present study was performed according to the Helsinki Declaration and approved by the Institutional Review Board (2022–058). All subjects have written informed consent.

Inclusion criteria: (1) age ≥ 40 years; (2) scoliosis with coronal Cobb angle ≥ 10°; (3) MRI/CT confirming lumbar stenosis; (4) failure of standardized conservative treatment; (5) planned UBE decompression alone (single‐ or multi‐level permitted); (6) completion of 18‐month follow‐up.

Exclusion criteria: (1) indication for fusion or deformity correction; (2) spondylolisthesis≥grade II or radiographic instability; (3) prior instrumented fusion; (3) active infection, tumor, or acute fracture; severe osteoporosis (T‐score ≤ −2.5) with recent fragility fracture; (4) intraoperative conversion to open surgery.

### 2.2 Surgical Technique

#### 2.2.1 Anesthesia and Positioning

After general anesthesia with endotracheal intubation, patients were placed prone on a radiolucent table with the abdomen free to lower venous pressure and reduce bleeding. Standard perioperative antibiotics, temperature control, and fluid management were applied. Continuous irrigation employed gravity flow with the fluid bag elevated 50 cm above the patient to maintain a clear endoscopic field.

#### 2.2.2 Operating Room Setup

In navigating procedures, an O‐arm/StealthStation intraoperative 3D navigation system (Medtronic, Minneapolis, MN, USA) was used. The endoscopic monitor was positioned on the operative side for the surgeon and assistant; the navigation display was placed at the patient's caudal end for easy line‐of‐sight.

#### 2.2.3 Reference Frame and Scanning

The reference frame was secured to the contralateral posterior superior iliac spine through a small stab incision/pin site, independent of the UBE portals. Stability of the frame was ensured throughout to prevent traction‐related drift. After draping, a baseline O‐arm CT was acquired, automatically transferred for multiplanar reconstruction and automatic registration, followed with instrument registration (navigation pointer, dilators, burr, UBE cannulas/handles). This workflow typically required less than 10 min. Re‐scanning was not routine and was only performed if navigation drift was suspected or when re‐verification was required at predefined milestones (e.g., after reaching the contralateral side or prior to final boundary confirmation).

### 2.3 Boundary Verification and Re‐Scan Policy

At predefined milestones (completion of ipsilateral laminotomy and over‐the‐top contralateral reach, confirmation of adequate lateral recess/foraminal decompression, or any suspected spatial disorientation), the navigation pointer was used to verify cranial/caudal and medial/lateral limits. If substantial anatomical change or potential drift was suspected, a small field of view scan was performed to reconfirm registration accuracy.

#### 2.3.1 Approach and Portal Creation

**FIGURE 1:**
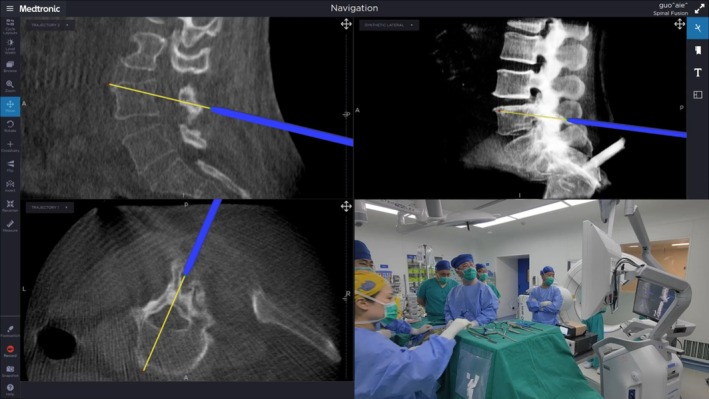
The navigation probe was used to plan the working channel for the unilateral biportal endoscopy.

With real‐time pointer guidance, skin projections and working trajectories were planned, defining the relative position and angulation of the viewing and working portals. Two longitudinal incisions (around 0.7–1.0 cm) were made lateral to the ipsilateral lamina at the target level (cranial portal for viewing; caudal portal for working). Cranio‐caudal spacing and portal angulation were adjusted to facilitate over‐the‐top access beneath the spinous process to the contralateral lamina and lateral recess. Under navigation, stepwise dilation was performed; the endoscopic sheath and working cannula were seated on the outer cortex of the target lamina (Figure 1). A SureTrak/universal tracker was affixed to the UBE handle or cannula to visualize depth and vector of the working corridor in real time.

#### 2.3.2 Navigation‐Assisted Decompression

Bipolar radiofrequency cleared fascia and soft tissue over the lamina, achieving meticulous hemostasis to preserve a stable underwater view. Using the navigation probe to delineate the interlaminar space and ligamentum flavum attachments, high‐speed burr and Kerrison rongeurs were used to perform incremental laminotomy and block‐segmental flavectomy, exposing the dural sac.

**FIGURE 2:**
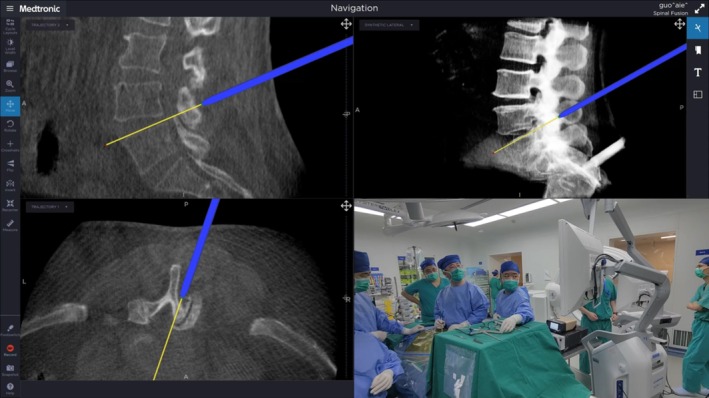
O‐arm navigation was used to accurately identify the anatomic location and range of decompression.

**FIGURE 3:**
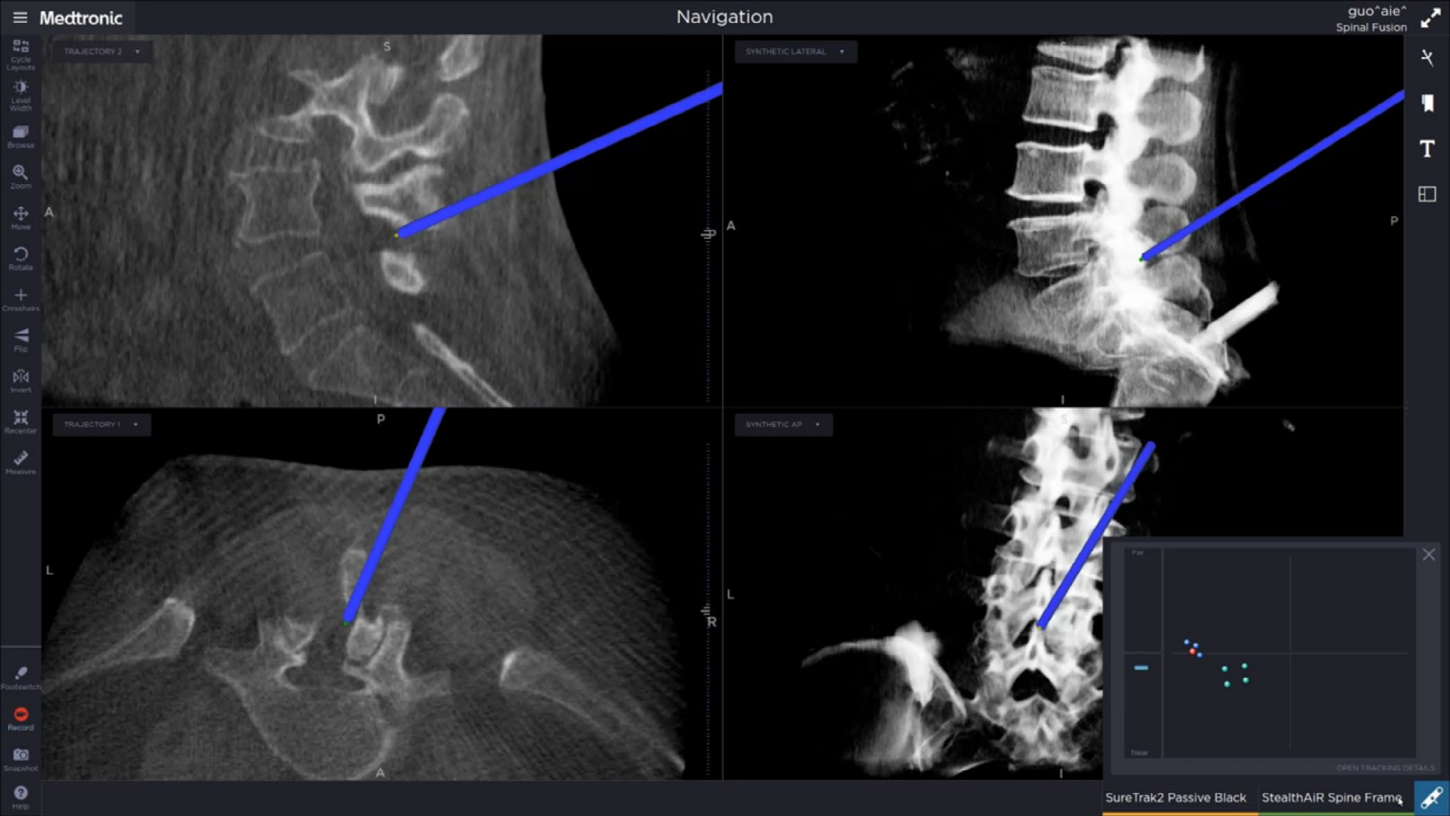
Accurate radiofrequency on surface of dural guided by O‐arm navigation.

Block‐segmental flavectomy was performed to remove the ligamentum flavum en bloc as a protective layer. After ipsilateral laminotomy with cranial/caudal edge undercutting, the ipsilateral flavum was sharply released from its attachments and removed as a single block whenever feasible; when markedly thickened or extensive, it was divided into two blocks to maintain controlled traction and reduce the risk of dural injury. Adequacy on the ipsilateral side was confirmed endoscopically by clear visualization of the dural sac margin and traversing root entry zone, with free dural pulsation and no residual compressive bands in the lateral recess. On the contralateral side, the flavum was dissected from the inner lamina and medial facet under navigation guidance and removed en bloc when possible; completion was defined by exposure of the contralateral dural border, a fully decompressed lateral recess, and unobstructed passage of a ball‐tip probe along the contralateral recess/foraminal entrance (Figure 2). Hemostasis was completed endoscopically, and a drain was placed as needed (Figure 3).

Quantitative facet undercutting was defined as navigation‐guided, boundary‐controlled medial facetectomy limited to the medial facet to achieve decompression while preserving the lateral facet column and capsule. The navigation probe was used to delineate the medial facet margin and plan the trajectory on multiplanar views, and burring was performed subcortically from the laminar undersurface toward the medial facet to enlarge the lateral recess and foraminal entrance without lateral column violation. Medial facetectomy was typically confined to the medial one‐third of the facet width.

#### 2.3.3 Usual UBE Decompression

In the NON‐NAV group, the same UBE workflow was performed, but all localization and boundary confirmation relied on C‐arm fluoroscopy and anatomical landmarks. Fluoroscopic checks confirmed cannula placement and burr trajectory at key steps; no intraoperative 3D navigation or re‐scanning was used. All procedures were performed by a single experienced endoscopic team.

### 2.4 Imaging Acquisition and 3D Quantification

#### 2.4.1 DCSA And Lateral Recess Metrics

**FIGURE 4:**
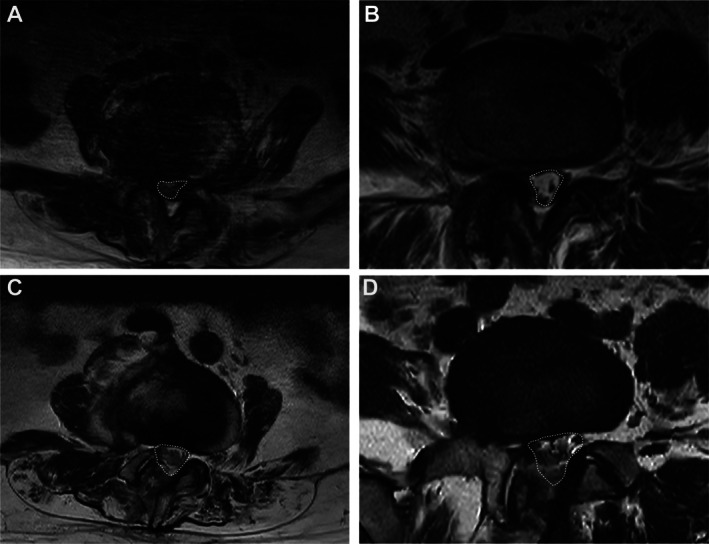
Preoperative and 1‐month postoperative axial T2‐weighted MRI at L4–5 showing DCSA changes. A, C: Non‐navigation (NON‐NAV), preoperative (A) and postoperative (C). (B, D) Navigation (NAV), preoperative (B) and postoperative (D).

**FIGURE 5:**
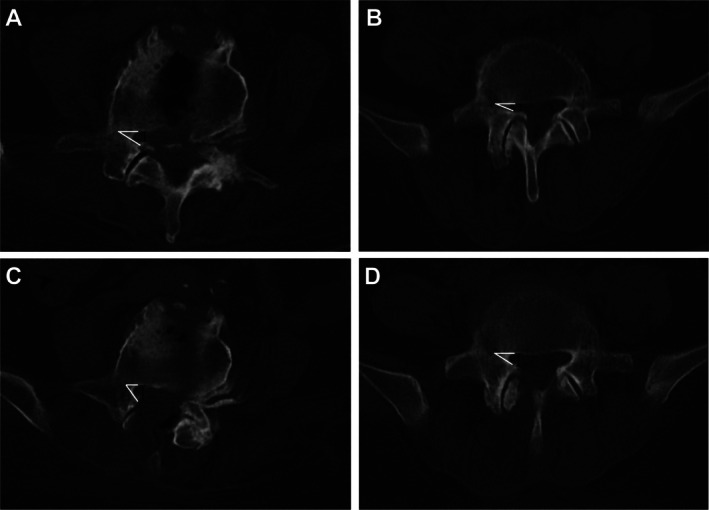
Preoperative and 1‐month postoperative axial CT at L4–5 showing lateral recess height and angle changes. (A, C) NON‐NAV, preoperative (A) and postoperative (C). (B, D) NAV, preoperative (B) and postoperative (D).

Preoperative axial T2‐weighted MRI and postoperative studies at 1 month and final follow‐up were co‐registered at the same level to measure the minimum DCSA [17] (Figure 4). Preoperative axial CT reconstructions and postoperative CT reconstructions at 1 month post‐op were co‐registered at the same level to measure lateral recess height/angle [18] (Figure 5A–D).

#### 2.4.2 3D Volumetry and Facet Preservation

Peri‐operative DICOM data of CT scanning (pre‐op and routine 1‐month post‐op; additional CT at the last follow‐up was included when available for exploratory analyses) was obtained to reconstruct the lamina–facet complex at the operated level. CT resolution was 0.35 × 0.35 mm per pixel and 2‐mm spacing between each axial slice. Volumetric analysis was performed using Materialize Mimics software (Materialize NV, Leuven, Belgium). Axial CT DICOMs (in‐plane resolution 0.35 × 0.35 mm; slice spacing 2.0 mm) were imported; regions of interest were manually delineated to segment the lamina and facet components. Volumes were computed as voxel count × in‐plane pixel spacing (LR × AP) × slice spacing (CC) obtained from DICOM metadata. The facet preservation rate (FPR) was calculated as FPR (%) = (postoperative lamina–facet volume)/(preoperative lamina–facet volume) × 100%, which was applied to report residual volumes and the percentage of bone resected20. Two raters independently segmented the lamina–facet complex. To assess inter‐rater reliability, intra‐class correlation coefficient was calculated and showed high consistency (0.846, 95% CI: 0.812–0.907).

Global alignment and segmental dynamics were assessed preoperatively, at 1 month, and at the final follow‐up: lumbar lordosis (LL), pelvic incidence (PI), PI–LL mismatch, coronal Cobb angles (L4–S1 and T12–S1), dynamic angulation (DA), and dynamic slip (DS).

### 2.5 Clinical Outcomes

Demographic and perioperative variables (age, sex, ASA classification, operated level, length of stay, operative time, estimated blood loss) were recorded. Clinical outcomes comprised visual analogue scale (VAS) scores and the Oswestry Disability Index (ODI), assessed preoperatively, 1 month postoperatively, and at the final follow‐up, along with the incidence of perioperative complications.

### 2.6 Statistical Analysis

Analyses were performed with IBM SPSS Statistics version 27.0 (IBM Corp., Armonk, NY, USA). Categorical variables were presented as frequencies and percentages, with between‐group comparisons conducted using the Chi‐squared test or Fisher's exact test, as appropriate. Continuous variables were first tested for normality using the Shapiro–Wilk test and for variance homogeneity using Levene's test. Normally distributed data were expressed as mean ± standard deviation (SD) and compared using independent‐samples *t*‐tests (Welch's *t*‐test applied if variances were unequal), whereas non‐normally distributed data were expressed as median (interquartile range) and analyzed using the Wilcoxon rank‐sum test. For repeated measures such as VAS scores, ODI, and radiographical parameters across time points, repeated‐measures ANOVA with post hoc pairwise comparisons (LSD or Tamhane's T2 depending on variance equality) was performed; if distributional assumptions were violated, the Friedman test was used. Effect sizes (mean differences or risk ratios) with 95% confidence intervals were reported alongside two‐tailed P values, with *p* < 0.05 considered statistically significant. In addition, exploratory Pearson correlation analyses were performed to examine associations between changes in clinical outcomes (ΔVAS/ΔODI) and radiological improvements (e.g., DCSA expansion, facet preservation ratio, and dynamic angulation/slip changes). Multivariable linear regression models with ΔODI as the dependent variable were also used to adjust for baseline ODI, age, and number of operated levels. To explore learning‐curve tendencies, operative time and estimated blood loss were additionally assessed against case order using linear regression.

## 3 Results

### 3.1 Patient Demographics and Perioperative Outcomes

A total of 63 radiographically stable ADS patients undergoing UBE decompression alone were analyzed: 34 in NAV group and 29 in NON‐NAV group. Baseline demographics and disease characteristics were comparable between groups, including age (66.8 ± 7.5 vs. 68.1 ± 6.0 years), sex (male 50.0% vs. 48.3%), ASA class (I/II/III: 38.2/47.1/14.7% vs. 31.0/51.7/17.2%), index level (L3–4: 11.8% vs. 24.1%; L4–5: 88.2% vs. 75.9%), and Schizas grade (C/D: 50.0/50.0% vs. 58.6/41.4%) (Table 1). Perioperative metrics were similar between NAV and NON‐NAV for operative time (90.1 ± 9.3 vs. 92.8 ± 8.7 min), estimated blood loss (66.4 ± 12.2 vs. 64.6 ± 13.7 mL), and length of stay (5.6 ± 1.7 vs. 5.9 ± 1.6 days). Follow‐up duration was slightly longer in NAV (21.2 ± 1.6 vs. 20.6 ± 1.6 months). Regarding adverse events, the NAV group had three incidental dural tears, all managed intraoperatively with endoscopic repair without postoperative neurological deficit. The NON‐NAV group had five dural tears and one superficial wound infection treated with antibiotics, with no reoperation required during follow‐up (Table 2). The last follow‐up assessment was defined as the final outpatient evaluation between 18 and 24 months postoperatively (overall mean follow‐up, 21.0 ± 1.6 months). To present the learning‐curve tendency, operative time and estimated blood loss were plotted against case order; no significant decreasing trend was observed (operative time: *β* = 0.12 min/case, *p* = 0.053; estimated blood loss: *β* = 0.03 mL/case, *p* = 0.74; Figures [Link], [Link]), which likely reflects that all procedures were performed by an experienced endoscopic team and that case complexity may vary across the series. Accordingly, the observed clinical and radiographic differences are less likely to be driven by temporal proficiency gains.

**TABLE 1:** Demographics and perioperative data.

Variable	NAV (*n* = 34)	NON‐NAV (*n* = 29)	*p*
Age (year)	66.8 ± 7.5	68.1 ± 6.0	0.467
Sex			1.000
Male	17	14	
Female	17	15	
BMI (kg/m^2^)	25.6 ± 3.2	25.3 ± 2.8	0.771
ASA PS classification grade			0.833
I	13	9	
II	16	15	
III	5	5	
Level			0.339
L3‐4	4	7	
L4‐5	30	22	
Schizas grade			0.667
C	17	17	
D	17	12	
Length of stay (day)	5.6 ± 1.7	5.9 ± 1.6	0.423
Operation time (min)	90.1 ± 9.3	92.8 ± 8.7	0.252
Estimated blood loss (mL)	66.4 ± 12.2	64.6 ± 13.7	0.579
Follow‐up (mo)	21.2 ± 1.6	20.6 ± 1.6	0.132

*Note:* Values are presented as mean ± standard deviation or number (%).

Abbreviations: ASA PS, American Society of Anesthesiologists physical status; NAV, O‐Arm Navigation‐Guided Unilateral Biportal Endoscopic surgery; NON‐NAV, Unilateral Biportal Endoscopic surgery.

**TABLE 2:** Clinical outcomes.

Variable	NAV (*n* = 34)	NON‐NAV (*n* = 29)	*p*
VAS (back)			
Pre‐Operative	7.1 ± 1.4	7.2 ± 1.4	0.673
1 Month	3.0 ± 0.8	3.2 ± 0.8	0.335
Last	1.1 ± 0.9	1.2 ± 0.7	0.653
VAS (leg)			
Pre‐Operative	7.6 ± 1.1	7.3 ± 1.3	0.361
1 Month	2.0 ± 0.9	2.1 ± 0.9	0.554
Last	1.7 ± 1.0	1.3 ± 1.0	0.119
ODI			
Preoperative	47.9 ± 9.2	47.2 ± 10.9	0.782
1 Month	19.6 ± 6.5	21.5 ± 5.5	0.225
Last	20.7 ± 5.7	22.3 ± 5.8	0.283
△VAS (back)			
1 Month ‐preoperative	−4.1 ± 1.7	−4.0 ± 1.6	0.897
Last ‐preoperative	−5.9 ± 1.5	−6.0 ± 1.6	0.891
△VAS (leg)			
1 Month ‐preoperative	−5.6 ± 1.5	−5.2 ± 1.7	0.317
Last ‐preoperative	−5.9 ± 1.6	−6.0 ± 1.4	0.759
△ODI			
1 Month ‐preoperative	−28.3 ± 12.0	−25.7 ± 11.6	0.392
Last ‐preoperative	−27.2 ± 9.6	−24.9 ± 11.9	0.404
Complication^a^			0.327
Yes	3	6	
No	31	23	

*Note:* Values are presented as mean ± standard deviation or number (%).

Abbreviations: ODI, Oswestry Disability Index; NAV, O‐arm navigation‐guided unilateral biportal endoscopic surgery; NIC, neurogenic intermittent claudication; NO‐NAV, unilateral biportal endoscopic surgery; VAS, visual analogue scal.

One‐way ANOVA, chi‐squared and Fisher's exact test were used for statistical analysis.

^a^ In NAV group, three cases of dura tear had occurred. In NO‐NAV group, five cases of dura tear and one case of infection had occurred.

### 3.2 Clinical Outcomes and Complications

Both groups experienced robust within‐group improvements in back‐pain VAS, leg‐pain VAS, and ODI at 1 month and at the last follow‐up relative to baseline; between‐group differences were not significant at either time point (e.g., back‐pain VAS at 1 month: 3.0 ± 0.8 vs. 3.2 ± 0.8, last: 1.1 ± 0.9 vs. 1.2 ± 0.7; leg‐pain VAS at 1 month: 2.0 ± 0.9 vs. 2.1 ± 0.9, last: 1.7 ± 1.0 vs. 1.3 ± 1.0; ODI at 1 month: 19.6 ± 6.5 vs. 21.5 ± 5.5, last: 20.7 ± 5.7 vs. 22.3 ± 5.8). The NAV group had a numerically lower complication rate (3/34) than the non‐NAV group (6/29), but this difference did not reach statistical significance on Fisher's exact test (Table 2). Clinical outcomes were evaluated preoperatively, at 1 month postoperatively, and at the last follow‐up time point (18–24 months).

### 3.3 Radiographic and 3D Volumetric Parameters

Global sagittal parameters (LL, PI, PI–LL mismatch) were comparable between groups at baseline and 1 month; at last follow‐up, PI–LL mismatch was slightly larger in NAV (2.7 ± 3.6 vs. 0.5° ± 3.6°, *p* = 0.021). Coronal Cobb angles (L4‐S1, T12‐S1) were likewise similar across time points, although L4‐S1 Cobb at last follow‐up was modestly larger in NAV (30.6° ± 4.5° vs. 28.3° ± 3.2°, *p* = 0.023), and the change in T12‐S1 Cobb at last follow‐up differed between groups (−0.2° ± 1.1° vs. 0.4 ± 1.0°, *p* = 0.022) (Table 3). At the segmental level, NON‐NAV exhibited greater increases in dynamic angulation and early dynamic slip (1‐month angulation 8.5° ± 1.2° vs. 6.2° ± 1.4°; last 9.3° ± 1.5° vs. 6.8° ± 1.5°; 1‐month slip 2.8 ± 0.8 vs. 1.8 ± 0.8 mm), with slip converging by the last visit (2.0 ± 1.1 vs. 2.0 ± 0.8 mm). Corresponding changes were likewise larger in NON‐NAV (dynamic change of angulation at 1 month 3.5 ± 0.3 vs. 1.0° ± 0.6°; last 4.3 ± 0.5 vs. 1.6° ± 0.8°; dynamic change of slip at 1 month 1.4 ± 0.3 vs. 0.5 ± 0.3 mm), although below the threshold for radiographic instability, the larger pre‐post changes in angulation and slip observed in the non‐navigation group may reflect less controlled decompressing boundaries (Table 3).

**TABLE 3:** Radiologic parameters.

Variable	NAV (*n* = 34)	NON‐NAV (*n* = 29)	*p*
Cobb angle L4S1			
Preoperative	29.8 ± 4.5	27.9 ± 3.5	0.077
1 Month	29.7 ± 4.5	28.1 ± 3.8	0.135
Last	30.6 ± 4.5	28.3 ± 3.2	0.023
Cobb angle T12S1			
Preoperative	41.9 ± 3.5	42.2 ± 3.4	0.792
1 Month	42.0 ± 3.6	42.1 ± 3.7	0.889
Last	41.7 ± 3.5	42.6 ± 3.4	0.329
PI (°)			
Preoperative	51.4 ± 6.0	49.2 ± 7.1	0.181
1 Month	51.3 ± 6.1	49.3 ± 7.1	0.236
Last	51.3 ± 6.4	48.5 ± 7.4	0.114
LL(°)			
Preoperative	50.5 ± 7.5	47.2 ± 8.8	0.114
1 Month	50.0 ± 8.3	47.7 ± 7.5	0.251
Last	48.6 ± 7.5	48.0 ± 8.4	0.766
PI‐LL mismatch (°)			
Preoperative	0.9 ± 3.8	2.0 ± 3.5	0.255
1 Month	1.2 ± 3.7	1.6 ± 2.8	0.688
Last	2.7 ± 3.6	0.5 ± 3.6	0.021
Dynamic angulation (°)			
Preoperative	5.2 ± 1.2	5.0 ± 1.4	0.579
1 Month	6.2 ± 1.4	8.5 ± 1.2	< 0.001*
Last	6.8 ± 1.5	9.3 ± 1.5	< 0.001*
Dynamic slip (mm)			
Preoperative	1.3 ± 0.8	1.3 ± 0.7	0.870
1 Month	1.8 ± 0.8	2.8 ± 0.8	< 0.001*
Last	2.0 ± 0.8	2.0 ± 1.1	0.713
DCSA (mm^2^)			
Preoperative	77.1 ± 12.7	79.3 ± 12.3	0.485
1 Month	230.1 ± 30.0	212.5 ± 34.9	0.036
Last	224.2 ± 32.0	204.6 ± 35.2	0.025*
Lateral recess height			
Preoperative	3.3 ± 1.5	2.7 ± 1.3	0.115
1 Month	13.4 ± 3.7	12.2 ± 4.7	0.227
Last	13.5 ± 3.7	12.2 ± 4.7	0.224
Lateral recess angle			
Preoperative	26.2 ± 4.6	28.0 ± 4.2	0.123
1 Month	55.4 ± 6.9	54.5 ± 11.1	0.710
Last	55.4 ± 7.1	54.8 ± 11.1	0.790
△Cobb angle L4S1			
1 Month	−0.0 ± 1.1	0.2 ± 1.1	0.398
Last	0.9 ± 1.4	0.4 ± 1.5	0.203
△Cobb angle T12S1			
1 Month	0.1 ± 1.2	−0.0 ± 1.2	0.737
Last	−0.2 ± 1.1	0.4 ± 1.0	0.022
△PI(°)			
1 Month	−0.1 ± 1.3	0.1 ± 1.0	0.410
Last	−0.1 ± 1.8	−0.7 ± 1.5	0.193
△LL(°)			
1 Month	−0.5 ± 5.7	0.5 ± 4.7	0.464
Last	−1.9 ± 6.0	0.8 ± 4.7	0.052
△PI‐LL mismatch(°)			
1 Month	0.3 ± 5.2	−0.4 ± 4.7	0.560
Last	1.4 ± 5.5	−1.1 ± 4.5	0.056
△Dynamic angulation (°)			
1 Month	1.0 ± 0.6	3.5 ± 0.3	< 0.001*
Last	1.6 ± 0.8	4.3 ± 0.5	< 0.001*
△Dynamic slip (mm)			
1 Month	0.5 ± 0.3	1.4 ± 0.3	< 0.001*
Last	0.8 ± 0.2	0.6 ± 0.6	0.301
△DCSA(mm^2^)			
1 Month	153.0 ± 24.5	133.1 ± 35.8	0.012
Last	147.1 ± 27.0	125.2 ± 36.0	0.008
△Lateral recess height			
1 Month	10.2 ± 3.1	9.4 ± 4.4	0.443
Last	10.2 ± 3.1	9.5 ± 4.4	0.442
△Lateral recess angle			
1 Month	29.1 ± 5.9	26.5 ± 10.0	0.203
Last	29.2 ± 6.1	26.8 ± 10.0	0.251
△Lamina–facet complex volume			
1 Month	−25.0 ± 3.6	−39.4 ± 3.3	< 0.001*
Last	−25.0 ± 3.9	−39.4 ± 3.5	< 0.001*
△Facet joint volume			
1 Month	−12.8 ± 2.4	−30.0 ± 2.9	< 0.001*
Last	−12.8 ± 2.7	−29.8 ± 2.8	< 0.001*

*Note:* CT‐based 3D volumetry (lamina–facet complex and facet joint volumes) was available for NAV *n* = 34 and NON‐NAV *n* = 28 due to one missing CT dataset in the NON‐NAV group. Values are presented as mean ± standard deviation or number (%). Independent‐samples *t* test (or Mann–Whitney *U* test when appropriate) and chi‐squared/Fisher's exact test were used for between‐group comparisons; within‐group changes over time were evaluated using repeated‐measures analysis as described in the Methods.

Abbreviations: DCSA, dural sac cross‐sectional area; LL, lumbar lordosis; NAV, O‐arm navigation‐guided unilateral biportal endoscopic surgery; NON‐NAV, Unilateral Biportal Endoscopic surgery; PI, pelvic incidence.

* *p* < 0.05, statistically significant differences.

### 3.4 Exploratory Correlation Analyses

Radiographically, DCSA increased markedly in both groups (~200%–250%). Absolute postoperative DCSA was larger in the NAV group (1 month: 230.1 ± 30.0 vs. 212.5 ± 34.9 mm^2^; last: 224.2 ± 32.0 vs. 204.6 ± 35.2 mm^2^), and the absolute increase in DCSA (△DCSA) was also greater in NAV (1 month: 153.0 ± 24.5 vs. 133.1 ± 35.8 mm^2^; last: 147.1 ± 27.0 vs. 125.2 ± 36.0 mm^2^) (Table 3). Importantly, between‐patient variance of DCSA was smaller in NAV (e.g., SD at 1 month, 30.01 vs. 34.85 mm^2^), indicating more consistent decompression (Table 3). Lateral recess height and angle improved in both groups with similar means at last follow‐up (height 13.5 ± 3.7 vs. 12.2 ± 4.7 mm; angle 55.4 ± 7.1 vs. 54.8° ± 11.1°), but they still showed larger variance in NON‐NAV. CT‐based 3D volumetry was available in 62 patients (NAV *n* = 34; NON‐NAV *n* = 28) due to one missing CT dataset in the NON‐NAV group. On CT‐based 3D volumetry, residual lamina–facet complex and residual facet volume were significantly higher in NAV at 1 month (75.0 ± 3.6 vs. 60.6 ± 3.3; 87.2 ± 2.4 vs. 70.0 ± 2.9, respectively), corresponding to smaller resection magnitudes (lamina–facet: −25.0 ± 3.6 vs. −39.4 ± 3.3; facet: −12.8 ± 2.4 vs. −30.0 ± 2.9) and confirming superior structural preservation with navigation (Table 3). Exploratory analyses were conducted to examine the relationship between clinical improvement and radiological changes at the last follow‐up. DCSA expansion ratio was not correlated with ODI improvement (*r* = −0.10, *p* = 0.43, Figure S3A) or leg‐pain improvement (*r* = −0.01, *p* = 0.92, Figure S3B), and FPR was not strongly correlated with ODI improvement (*r* = 0.14, *p* = 0.29, Figure S3C). By contrast, greater absolute change in dynamic angulation (|ΔDA|) was associated with less ODI improvement (r = −0.26, *p* = 0.041, Figure S3D).

## 4 Discussion

### 4.1 Clinical Improvement and Perioperative Outcomes

In this cohort of radiographically stable adult degenerative scoliosis (ADS), we compared UBE decompression alone assisted with O‐arm navigation against conventional fluoroscopy‐guided UBE. We observed comparable improvements in symptom and function between groups. Perioperatively, operative time was slightly shorter in the navigation group, while estimated blood loss and length of stay were similar. Although navigation improved structural preservation, the between‐group differences in VAS/ODI did not reach statistical significance. A plausible explanation is that both techniques achieved adequate decompression for symptom relief in radiographically stable ADS, producing a ceiling effect for short‐term outcomes. Global sagittal parameters did not differ between groups at baseline or across the two postoperative assessments. At the segmental level, however, the non‐navigation group exhibited greater pre–post changes in angulation and anteroposterior slip. Although these changes did not exceed radiographic thresholds for instability, they suggest a tendency toward over‐resection without navigation. Collectively, our findings align with prior evidence indicating that, in stable ADS, UBE decompression alone can yield acceptable symptomatic benefit [19], while O‐arm navigation exhibits superiority in accurate decompression and facet preservation.

### 4.2 Decompression Precision and Boundary Control

Quantitative imaging literature commonly cites DCSA < 100 mm^2^ as a diagnostic or adequacy threshold for lumbar stenosis [18, 20, 21]. Importantly, several clinical studies indicate that once adequacy is reached, further canal enlargement does not linearly translate into greater symptomatic relief [18, 20, 21]. Mechanistically, both groups achieved robust expansion of DCSA for approximately 200%–250%, and comparable decompression of lateral recess; however, the dispersion of DCSA change was smaller in NAV group. Thus, navigation did not produce better outcomes by excess decompression, but rather by conferring more controllable boundaries, better structural preservation, and more tempered biomechanical perturbation. With navigation, the distribution of pathology (central canal, lateral recess) and key osseous landmarks (hypertrophic facets) are explicitly externalized, enabling targeted, adequate decompression in the highly variable anatomy of ADS and reducing inadvertent over‐resection when endoscopic orientation is challenging [14]. The relationship between radiographic improvement and patient‐reported outcomes is not necessarily linear. Clinical outcomes such as VAS and ODI capture patient‐perceived pain and disability, which are influenced not only by the degree of static canal enlargement but also by time‐dependent neural recovery, postoperative inflammation, rehabilitation, and the presence of heterogeneous pain generators (e.g., facetogenic or myofascial pain) that may persist after adequate decompression. Accordingly, once a sufficient decompression threshold is reached, additional increases in canal area may produce diminishing returns in symptoms and function. This interpretation is supported by clinical evidence showing that postoperative DCSA is not clearly associated with outcome after lumbar stenosis surgery [20], and is consistent with our observation of comparable VAS/ODI improvement despite measurable differences in DCSA expansion and structural preservation metrics. In our exploratory analyses, DCSA expansion at last follow‐up was not correlated with ODI improvement (*r* = −0.10, *p* = 0.43) or leg‐pain improvement (*r* = −0.01, *p* = 0.92), and facet preservation ratio (FPR) was also not strongly correlated with ODI improvement (*r* = 0.14, *p* = 0.29). By contrast, larger absolute changes in dynamic angulation (|ΔDA|) were associated with less ODI improvement (*r* = −0.26, *p* = 0.041), and this association remained borderline after adjustment for baseline ODI, age, and number of operated levels (*β* = −1.97 ODI points per 1° increase in |ΔDA|, *p* = 0.050). These findings suggest that, once decompression is sufficient, postoperative motion perturbation may be more closely related to functional recovery than static canal‐area enlargement.

### 4.3 Facet Preservation and Segmental Stability

The struggle to find balance between facetectomy and preservation of segmental stability is essential in UBE decompression. Graded facetectomy experiments show that medial/partial resection has limited impact on stability, whereas > 50% resection substantially increases angular and shear displacement; precise control of the resection boundary is therefore critical [22, 23, 24]. In the present study, with equivalent neural decompression achieved, the navigation group demonstrated a higher and more tightly distributed facet preservation rate (FPR) and residual lamina–facet volume. Consistently, the non‐navigation group exhibited larger changes in angulation and slip from the pre‐ to post‐operative states (particularly at high‐motion segments such as L4/5) which, while not crossing formal instability thresholds, are suggestive of excessive segmental release. Recent technical reports and case series integrating O‐arm navigation with UBE indicate that navigation provides real‐time spatial feedback during portal creation, burr trajectory planning, and bilateral corridor access, thereby reducing the risk of over‐ or under‐facetectomy [16]. This promotes visualized stopping rules and reproducible, individualized precision decompression, offering a more robust solution to the paradox in treating ADS: adequate decompression while maintaining stability [13, 15].

### 4.4 Surgical Efficiency and Safety Profile

From a workflow standpoint, our finding of a slightly shorter operative time with navigation is consistent with the literature on the UBE learning curve and efficiency [25]. UBE exhibits a clear learning curve, with meta‐analysis suggesting a plateauing of operative time and complications after approximately 38 cases [6]. On this basis, standardized registration, pathway planning, and team choreography with O‐arm navigation may not necessarily prolong procedures. Instead, it may offset setup costs by reducing spatial disorientation, minimizing repeated fluoroscopy [26]. Regarding radiation, navigation may lower staff exposure (longer distance from the source), whereas patient dose depends on scanning strategy and adherence to low‐dose protocols; as always, a balance between accuracy and dose is required [11, 27].

In our cohort, the overall complication burden was low and statistically similar between groups; nonetheless, the navigation cohort had 3 dural tears, whereas the non‐navigation cohort experienced five dural tears and one superficial wound infection. These event counts align with contemporary endoscopic literature, in which dural tear is the most frequent adverse event after UBE decompression, typically ranging around 2.5%–4.5% across meta‐analyses and pooled series [28]. Importantly, ADS and advanced degenerative stenosis concentrate several known DT (dural tear) risk factors—older age, severe/bilateral stenosis, facet hypertrophy/ossification, and sometimes revision scarring—which have been consistently linked to higher dural tear risk in lumbar decompression and in adult deformity populations [29, 30]. In the endoscopic setting, continuous saline irrigation adds a unique hazard: once a dural tear occurs, hydrostatic pressure can facilitate fluid/air entry into the thecal space, amplifying neurological and intracranial sequelae if not promptly controlled [31]. Taken together, these factors help explain why UBE decompression in ADS can be complication‐prone despite minimally invasive access.

From a technical standpoint, O‐arm navigation may mitigate these risks via real‐time 3D orientation, explicit visualization of osseous landmarks and confirmation of cranial/caudal and medial/lateral boundaries, thereby reducing “trial‐and‐error” drilling near the flavum–dura interface [32]. Although direct RCT‐level evidence for a reduction in endoscopic dural tear with navigation is limited, navigation has repeatedly improved localization accuracy and instrument trajectory control in decompression and instrumented procedures; these mechanisms are plausibly transferable to UBE in rotated, tortuous ADS anatomy [33]. Our numerically lower event count in the NAV group (with no infections) is consistent with this risk‐attenuation hypothesis, even if underpowered for rare‐event statistics.

### 4.5 Study Strengths and Limitations

Despite its retrospective nature, the present study has several notable strengths. First, it is one of the few comparative studies to specifically evaluate the utility of O‐arm navigation in UBE decompression within the highly tortuous and variable anatomical environment of adult degenerative scoliosis. Second, the incorporation of CT‐based 3D volumetry provides a highly precise, objective quantification of the lamina–facet complex, offering more robust evidence for structural preservation than conventional 2D radiographic measurements. Finally, all surgical procedures were performed by a single experienced endoscopic team, which effectively minimized confounding baseline variables related to varying surgeon proficiency and ensured a standardized surgical technique across both cohorts.

The present study has several limitations: first, its single‐center, retrospective design cannot fully eliminate selection bias and residual confounding; as all procedures were performed by a single experienced endoscopic team, a surgeon‐experience stratification was not applicable, which may limit the generalizability of our findings to centers with varying levels of proficiency; second, segmentation and registration errors in imaging‐based quantification cannot be entirely excluded; third, the 24‐month follow‐up may be underpowered to detect late instability, adjacent‐segment load transfer, or reoperation events; finally, the cost–benefit trade‐offs were not formally modeled. Additionally, patient radiation dose metrics (e.g., CTDIvol/DLP) and the exact number of O‐arm re‐scans were not consistently retrievable in this retrospective cohort. In addition, because navigation was implemented during an evolving endoscopic program, temporal trends and learning effects could confound the association between navigation and structural preservation. Although operative time did not increase with navigation, future studies should incorporate calendar‐time adjustment or case‐sequence stratification to isolate the causal effect of navigation from maturing team proficiency. In addition, the present sample size was primarily powered around the CT‐based structural endpoint rather than detecting subtle between‐group PROM differences, which may require larger cohorts to confirm. Future work should proceed along three lines: (i) multicenter prospective (ideally randomized) comparisons to test medium or long‐term safety and effectiveness of navigation; (ii) development of a precision‐to‐prognosis bridge model that aggregates 3D structural parameters of lamina‐facet resection boundaries into a precision index, links it to functional recovery, and quantifies its clinical utility; and (iii) optimization of minimal‐necessary scanning strategies, comparing single registration versus staged verification in terms of dose‐precision marginal gains, and defining an actionable safe window for facet preservation, particularly at high‐motion levels such as L4/5 segment.

## 5 Conclusion

In patients with radiographically stable adult degenerative scoliosis, O‐arm navigation–assisted unilateral biportal endoscopic decompression achieves the same clinical improvement as conventional techniques, without prolonging operative time or increasing blood loss. Navigation provides more precise decompression with higher facet preservation and reduced segmental perturbation, thereby lowering the risk of postoperative instability. These findings support the use of navigation in selected patients to enhance surgical safety and long‐term stability.

## Author Contributions

**Yi Liu:** conceptualization, data curation, investigation, validation, writing – original draft, formal analysis. **Yiwei Xie:** formal analysis, data curation, methodology, investigation, software. **Zhibao Chen:** software, data curation, validation, investigation. **Ruijun Xu:** supervision, resources, data curation, software. **Haojie Chen:** software, visualization, validation, resources. **Xiaojian Ye:** conceptualization, investigation, writing – review and editing, project administration, supervision, resources. **Jiangming Yu:** funding acquisition, project administration, conceptualization, methodology, supervision, resources, writing – review and editing, visualization.

## Funding

This study was supported by the Laboratory Open Fund of Key Technology and Materials in Minimally Invasive Spine Surgery (Grant No. 2024JZWC‐YBA02), Medical Research Project of Shanghai Changning Health Commission (Grant No. 2024QN04).

## Ethics Statement

This study protocol was approved by the Ethics Committee of Tongren Hospital, Shanghai Jiao Tong University School of Medicine before data collection and analysis (Approval No. 2022–058).

## Conflicts of Interest

The authors declare no conflicts of interest.

## Supporting information


**Figure S1:** Case order versus operative time. Scatter plot showing operative time (minutes) plotted against chronological case order for the entire cohort. The fitted linear regression line is shown.

**Figure S2:** Case order versus estimated blood loss. Scatter plot showing estimated blood loss (mL) plotted against chronological case order for the entire cohort. The fitted linear regression line is shown.

**Figure S3:** Associations between clinical improvement and radiological changes at the last follow‐up. (A) ODI improvement (Pre − Last) versus DCSA expansion ratio (Last/Pre). (B) Leg‐pain VAS improvement (Pre − Last) versus DCSA expansion ratio (Last/Pre). (C) ODI improvement (Pre − Last) versus CT‐based facet preservation ratio at last follow‐up (% of baseline). (D) ODI improvement (Pre − Last) versus absolute change in dynamic angulation at last follow‐up (|ΔDA|, degrees). Pearson's correlation coefficient (r), two‐tailed P value, and sample size (N) are displayed in each panel, with a fitted linear regression line.

## Data Availability

The datasets generated during and/or analyzed during the current study are available from the corresponding author on reasonable request.

## References

[1] J. McAviney , C. Roberts , B. Sullivan , A. J. Alevras , P. L. Graham , and B. T. Brown , “The Prevalence of Adult de Novo Scoliosis: A Systematic Review and Meta‐Analysis,” European Spine Journal 29, no. 12 (2020): 2960–2969. 32440771 10.1007/s00586-020-06453-0

[2] A. Kelly and A. Younus , “Adult degenerative scoliosis – A literature review,” Interdisciplinary Neurosurgery: Advanced Techniques and Case Management 20 (2020): 100661.

[3] J.‐w. Kwon , S.‐H. Moon , S.‐Y. Park , et al., “Lumbar Spinal Stenosis: Review Update 2022,” Asian Spine Journal 16, no. 5 (2022): 789–798. 36266248 10.31616/asj.2022.0366 PMC9633250

[4] H. Wang , X. Liu , Y. Li , et al., “The Selection of a Surgical Strategy for the Treatment of Adult Degenerative Scoliosis With “Pear‐Shaped” Decompression Under Open Spinal Endoscopy,” Scientific Reports 14, no. 1 (2024): 16019. 38992132 10.1038/s41598-024-67003-y PMC11239948

[5] S. M. Park , K. S. Song , D. W. Ham , et al., “Safety Profile of Biportal Endoscopic Spine Surgery Compared to Conventional Microscopic Approach: A Pooled Analysis of 2 Randomized Controlled Trials,” Neurospine 21, no. 4 (2024): 1190–1198. 39765264 10.14245/ns.2448718.359 PMC11744543

[6] S. X. Liu , R. S. Chen , C. M. Chen , L. R. He , S. W. Jhang , and G. X. Lin , “Unilateral Biportal Endoscopic Spine Surgery: A Meta‐Analysis Unveiling the Learning Curve and Clinical Benefits,” Frontiers in Surgery 11 (2024): 1405519. 39575448 10.3389/fsurg.2024.1405519 PMC11578948

[7] J. Xu , D. Wang , J. Liu , et al., “Learning Curve and Complications of Unilateral Biportal Endoscopy: Cumulative Sum and Risk‐Adjusted Cumulative Sum Analysis,” Neurospine 19, no. 3 (2022): 792–804. 35996762 10.14245/ns.2143116.558 PMC9537833

[8] H. Wang , X. Li , B. Li , et al., “Analysis of the Learning Curve for Unilateral Biportal Endoscopic Technique Using CUSUM Method on Fresh Frozen Cadavers,” BMC Musculoskeletal Disorders 25, no. 1 (2024): 1007. 39643896 10.1186/s12891-024-08123-4 PMC11624593

[9] Y. Chen , W. Lin , S. Lei , et al., “Comparing the Efficacy and Safety of Unilateral Biportal Endoscopic Decompression With Percutaneous Endoscopic Lumbar Decompression for Lumbar Degenerative Diseases: A Meta‐Analysis,” World Neurosurgery 187 (2024): e383–e398. 38657790 10.1016/j.wneu.2024.04.093

[10] N. Rawicki , J. E. Dowdell , and H. S. Sandhu , “Current State of Navigation in Spine Surgery,” Annals of Translational Medicine 9, no. 1 (2021): 85. 33553378 10.21037/atm-20-1335 PMC7859779

[11] S. Virk and S. Qureshi , “Navigation in Minimally Invasive Spine Surgery,” Journal of Spine Surgery 5 (2019): S25–S30. 31380490 10.21037/jss.2019.04.23 PMC6626747

[12] Y.‐S. Lee , D.‐C. Cho , and K.‐T. Kim , “Navigation‐Guided/Robot‐Assisted Spinal Surgery: A Review Article,” Neurospine 21, no. 1 (2024): 8–17. 38569627 10.14245/ns.2347184.592 PMC10992634

[13] J. Quillo‐Olvera , J. Quillo‐Reséndiz , D. Quillo‐Olvera , M. Barrera‐Arreola , and J. S. Kim , “Ten‐Step Biportal Endoscopic Transforaminal Lumbar Interbody Fusion Under Computed Tomography‐Based Intraoperative Navigation: Technical Report and Preliminary Outcomes in Mexico,” Operative Neurosurgery 19, no. 5 (2020): 608–618. 32726423 10.1093/ons/opaa226

[14] X. Huang , J. Gong , H. Liu , et al., “Unilateral Biportal Endoscopic Lumbar Interbody Fusion Assisted by Intraoperative O‐Arm Total Navigation for Lumbar Degenerative Disease: A Retrospective Study,” Frontiers in Surgery 9 (2022): 1026952. 36211257 10.3389/fsurg.2022.1026952 PMC9539070

[15] D. H. Lee , C. K. Park , J. S. Kim , et al., “O‐Arm Navigation‐Based Transforaminal Unilateral Biportal Endoscopic Discectomy for Upper Lumbar Disc Herniation: An Innovative Preliminary Study,” Asian Spine Journal 19, no. 2 (2025): 194–204. 40195630 10.31616/asj.2025.0072 PMC12061600

[16] R. A. Kavishwar , Y. Liang , D. Lee , J. Kim , M. Pedraza , and J. S. Kim , “O‐Arm Navigation‐Guided Unilateral Biportal Endoscopic Decompression of Far‐Out Syndrome,” Neurospine 21, no. 4 (2024): 1149–1153. 39765258 10.14245/ns.2449140.570 PMC11744545

[17] M. H. Lee , H. J. Jang , B. J. Moon , et al., “Comparative Outcomes of Biportal Endoscopic Decompression, Conventional Subtotal Laminectomy, and Minimally Invasive Transforaminal Lumbar Interbody Fusion for Lumbar Central Stenosis,” Neurospine 21, no. 4 (2024): 1178–1189. 39765263 10.14245/ns.2448830.415 PMC11744548

[18] J. Steurer , S. Roner , R. Gnannt , and J. Hodler , “Quantitative Radiologic Criteria for the Diagnosis of Lumbar Spinal Stenosis: A Systematic Literature Review,” BMC Musculoskeletal Disorders 12 (2011): 175. 21798008 10.1186/1471-2474-12-175 PMC3161920

[19] M. Echt , R. De la Garza Ramos , E. Geng , et al., “Decompression Alone in the Setting of Adult Degenerative Lumbar Scoliosis and Stenosis: A Systematic Review and Meta‐Analysis,” Global Spine Journal 13, no. 3 (2023): 861–872. 36127159 10.1177/21925682221127955 PMC10240589

[20] E. Hermansen , T. Myklebust , C. Weber , et al., “Postoperative Dural Sac Cross‐Sectional Area as an Association for Outcome After Surgery for Lumbar Spinal Stenosis: Clinical and Radiological Results From the NORDSTEN‐Spinal Stenosis Trial,” Spine (Phila Pa 1976) 48, no. 10 (2023): 688–694. 36809364 10.1097/BRS.0000000000004565 PMC10118242

[21] T. Strojnik , “Measurement of the Lateral Recess Angle as a Possible Alternative for Evaluation of the Lateral Recess Stenosis on a CT Scan,” Wiener Klinische Wochenschrift 113, no. Suppl 3 (2001): 53–58. 15503622

[22] K. Abumi , M. M. Panjabi , K. M. Kramer , J. Duranceau , T. Oxland , and J. J. Crisco , “Biomechanical Evaluation of Lumbar Spinal Stability After Graded Facetectomies,” Spine (Phila Pa 1976) 15, no. 11 (1990): 1142–1147. 2267608 10.1097/00007632-199011010-00011

[23] Z. L. Zeng , R. Zhu , Y. C. Wu , et al., “Effect of Graded Facetectomy on Lumbar Biomechanics,” Journal of Healthcare Engineering 2017 (2017): 7981513. 29065645 10.1155/2017/7981513 PMC5337791

[24] S. Ahuja , A. N. Moideen , A. G. Dudhniwala , E. Karatsis , L. Papadakis , and E. Varitis , “Lumbar Stability Following Graded Unilateral and Bilateral Facetectomy: A Finite Element Model Study,” Clinical Biomechanics (Bristol) 75 (2020): 105011. 10.1016/j.clinbiomech.2020.105011 32335473

[25] K. T. Chen , M. S. Song , and J. S. Kim , “How I Do It? Interlaminar Contralateral Endoscopic Lumbar Foraminotomy Assisted With the O‐Arm Navigation,” Acta Neurochirurgica 162, no. 1 (2020): 121–125. 31811466 10.1007/s00701-019-04104-y

[26] K. Wu , Z. Yun , S. Suvithayasiri , et al., “Evolving Paradigms in Spinal Surgery: A Systematic Review of the Learning Curves in Minimally Invasive Spine Techniques,” Neurospine 21, no. 4 (2024): 1251–1275. 39765270 10.14245/ns.2448838.419 PMC11744536

[27] R. Alshaibi , A. A. Mohamed , C. Williams , and B. Lucke‐Wold , “Exoscope Visualization, Navigation Guidance, and Robotic Precision in Spine Surgery,” Journal of Minimally Invasive Spine Surgery and Technique 10, no. 1 (2025): 22–33.

[28] B. Wang , P. He , X. Liu , Z. Wu , and B. Xu , “Complications of Unilateral Biportal Endoscopic Spinal Surgery for Lumbar Spinal Stenosis: A Systematic Review of the Literature and Meta‐Analysis of Single‐Arm Studies,” Orthopaedic Surgery 15, no. 1 (2023): 3–15. 36394088 10.1111/os.13437 PMC9837251

[29] Z. A. F. Alshameeri and V. Jasani , “Risk Factors for Accidental Dural Tears in Spinal Surgery,” International Journal of Spine Surgery 15, no. 3 (2021): 536–548. 33986000 10.14444/8082 PMC8176817

[30] G. Toci , M. J. Lambrechts , T. Issa , et al., “Incidence, Risk Factors, and Outcomes of Incidental Durotomy During Lumbar Spine Decompression With or Without Fusion,” Asian Spine Journal 17, no. 4 (2023): 647–655. 37226383 10.31616/asj.2022.0297 PMC10460661

[31] J. L. Pao , “Preliminary Clinical and Radiological Outcomes of the “no‐Punch” Decompression Techniques for Unilateral Biportal Endoscopic Spine Surgery,” Neurospine 21, no. 2 (2024): 732–741. 38955542 10.14245/ns.2448376.188 PMC11224751

[32] A. K. Sharma , R. G. de Oliveira , S. Suvithayasiri , et al., “The Utilization of Navigation and Emerging Technologies With Endoscopic Spine Surgery: A Narrative Review,” Neurospine 22, no. 1 (2025): 105–117. 40211520 10.14245/ns.2449404.702 PMC12010863

[33] B. T. Wen , Z. Q. Chen , C. G. Sun , et al., “Three‐Dimensional Navigation (O‐Arm) Versus Fluoroscopy in the Treatment of Thoracic Spinal Stenosis With Ultrasonic Bone Curette: A Retrospective Comparative Study,” Medicine (Baltimore) 98, no. 20 (2019): e15647. 31096488 10.1097/MD.0000000000015647 PMC6531158

