# Supplementary figures and images for: O‐Arm Navigation Enhances Facet Preservation Without Compromising Clinical Outcomes in UBE Decompression for Radiographically Stable Adult Degenerative Scoliosis: A Single‐Center Comparative Study
Source: Orthop Surg. 2026 May 8;18(6):1203–15. doi: 10.1111/os.70315 (PMC13238630; doi:10.1111/os.70315)

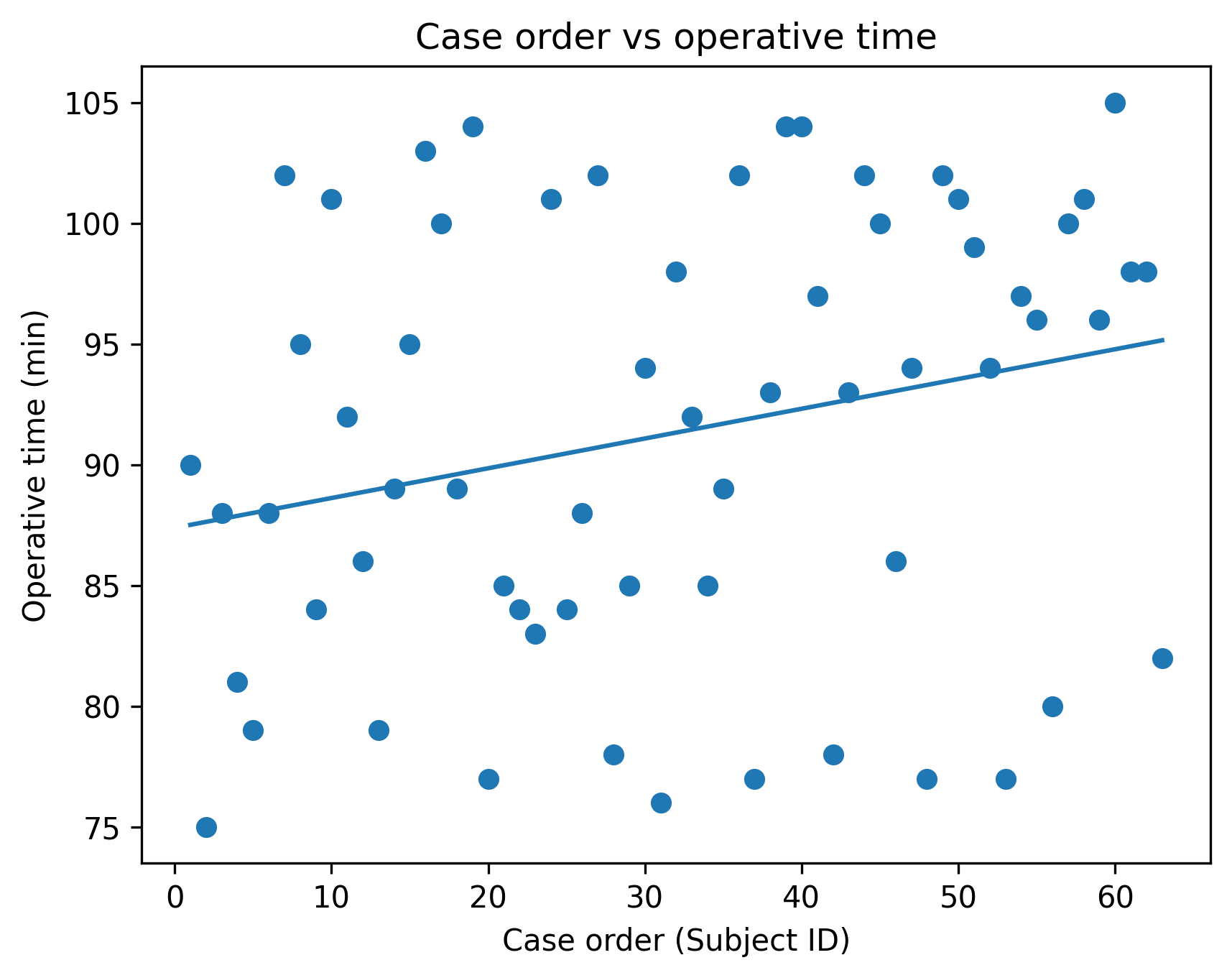

Supplement: Supplementary file 1 — Figure S1: Case order versus operative time. Scatter plot showing operative time (minutes) plotted against chronological case order for the entire cohort. The fitted linear regression line is shown. [file OS-18-1203-s002.png]

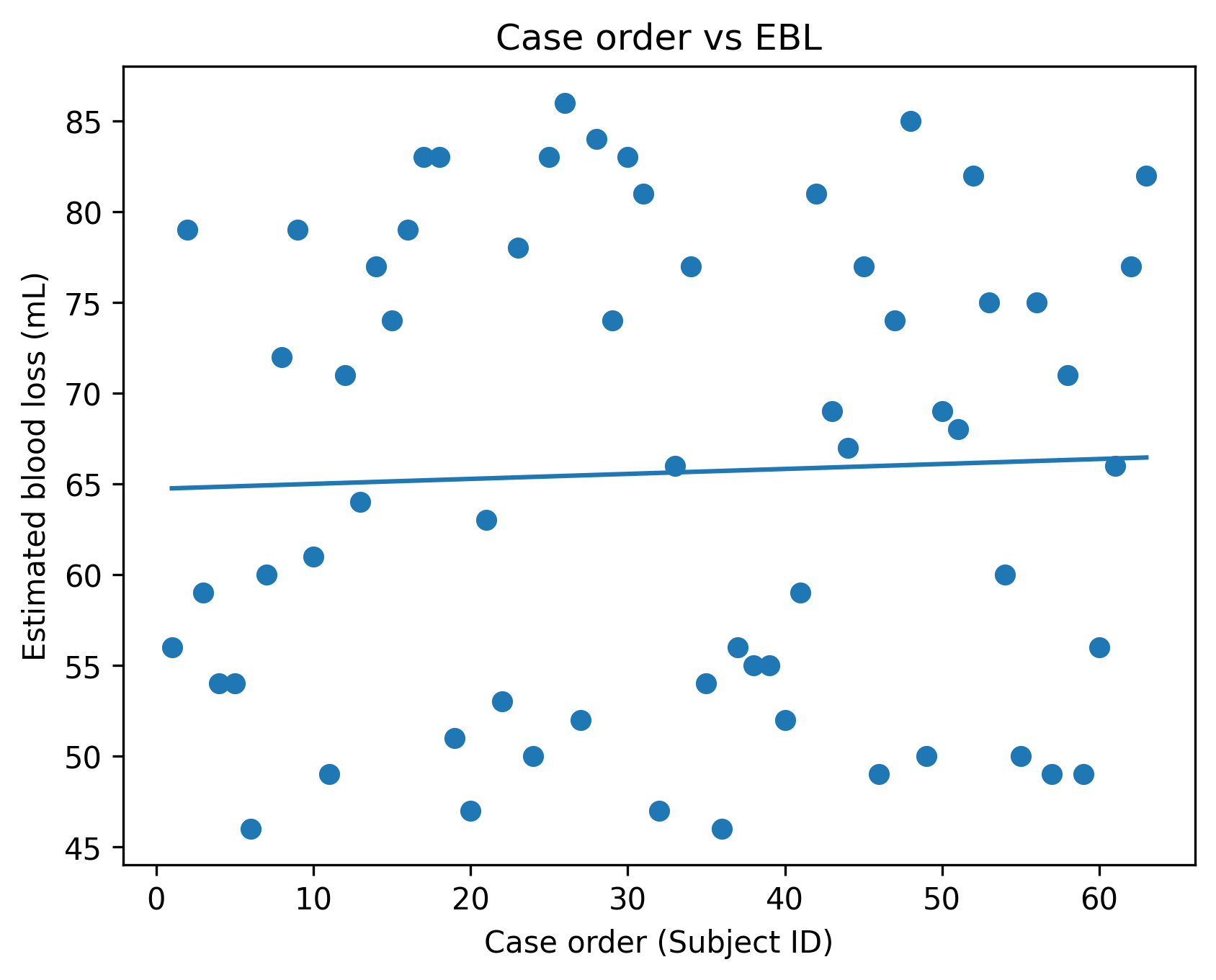

Supplement: Supplementary file 2 — Figure S2: Case order versus estimated blood loss. Scatter plot showing estimated blood loss (mL) plotted against chronological case order for the entire cohort. The fitted linear regression line is shown. [file OS-18-1203-s003.png]

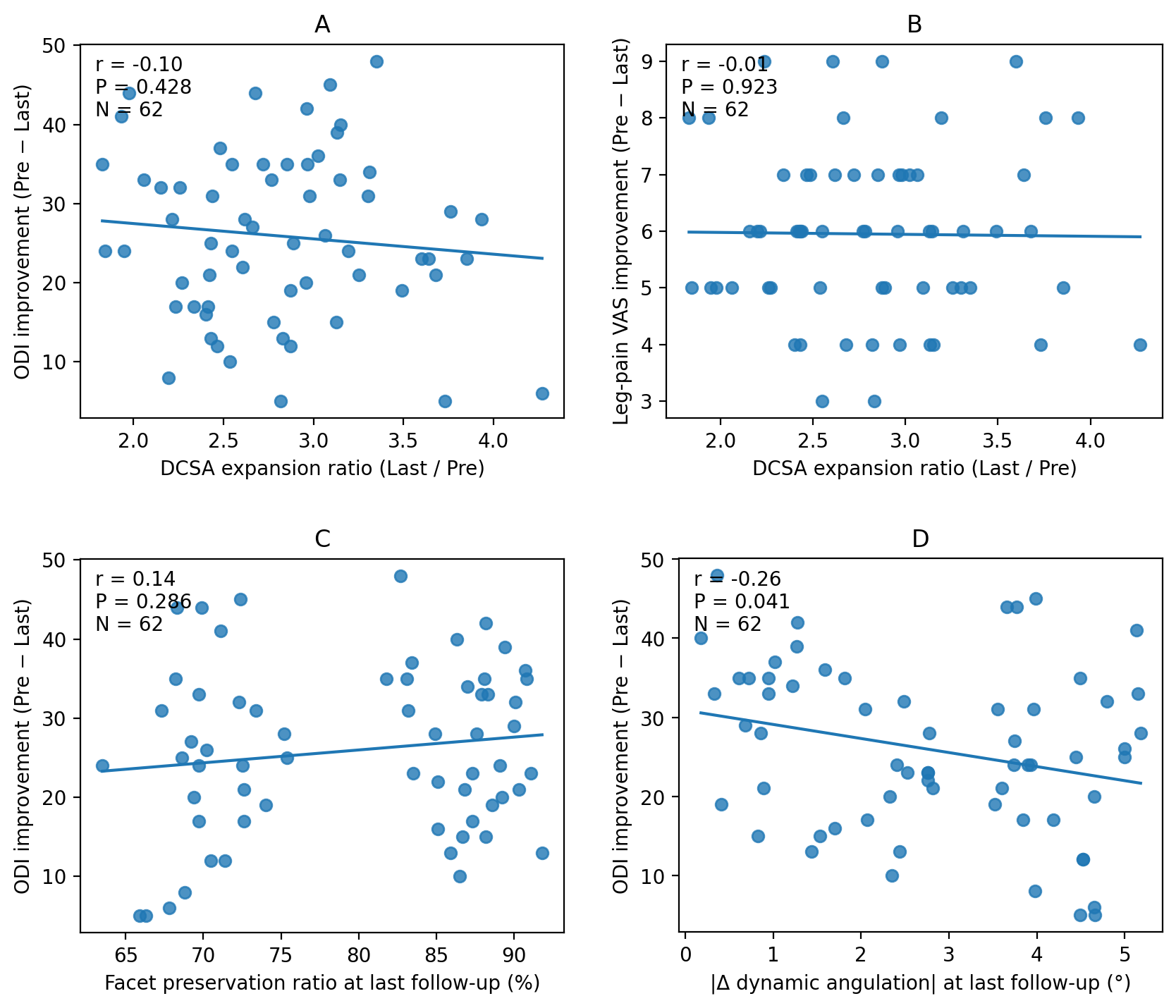

Supplement: Supplementary file 3 — Figure S3: Associations between clinical improvement and radiological changes at the last follow‐up. (A) ODI improvement (Pre − Last) versus DCSA expansion ratio (Last/Pre). (B) Leg‐pain VAS improvement (Pre − Last) versus DCSA expansion ratio (Last/Pre). (C) ODI improvement (Pre − Last) versus CT‐based facet preservation ratio at last follow‐up (% of baseline). (D) ODI improvement (Pre − Last) versus absolute change in dynamic angulation at last follow‐up (|ΔDA|, degrees). Pearson's correlation coefficient (r), two‐tailed P value, and sample size (N) are displayed in each panel, with a fitted linear regression line. [file OS-18-1203-s001.png]
